# The grapefruit: an old wine in a new glass? Metabolic and cardiovascular perspectives

**Published:** 2012-06

**Authors:** PMO Owira

## Dear Sir

The letter by Mullier^1^ in response to our article titled ‘The grapefruit: an old wine in a new glass? Metabolic and cardiovascular perspectives’^2^ refers. The author states that amiodarone is not only a prodrug but also has inherent pharmacodynamic effects, just like its metabolite N-desethyamiodarone (N-DEA), which he correctly suggests could have even greater pharmacological effects than the parent compound. However, we need to emphasise that even though N-DEA has similar class III anti-arrythmic effects, it has faster sodium channel blockade and lower class IV effects than amiodarone.^3^-^8^

The inhibition of pre-hepatic/hepatic CYP3A4 metabolism of amiodarone alters both plasma and cardiac substrate:metabolite ratios. It therefore reduces alterations of PR and QT_C_ intervals,^9^ and hence diminishes the anti-arrythmic effects of amiodarone. Both amiodarone and N-DEA have long half-lives (50 and 60 days, respectively),^10^-^12^ and at normal therapeutic doses, the relative contribution of either to the anti-arrythmic and overall cardiac electrophysiological effects is not presently known, despite the aforementioned interaction with grapefruit juice. This, however, does not disqualify amiodarone as a prodrug.

The interaction of grapefruit juice with amiodarone is more complicated than previously thought. Naringenin, the naringin (the predominant flavonoid in grapefruit juice) aglycone, has recently been reported to prolong QT_C_ by inhibiting the rapid component of delayed rectifier K^+^ current (*I*_kr_), leading to significant QT prolongation in healthy subjects and in patients with dilated or hypertensive cardiomyopathy,^13^ as well as in experimental conditions.^14^ It is therefore envisaged that the pro-arrythmic actions of naringin or grapefruit juice, just like all class III anti-arrythmic agents, may put patients with myocardial structural disorders at risk of provoking torsades des pointes.

Even though cases of QT prolongation and torsades de pointes with amiodarone are rare, a case has been reported of a female patient who presented with marked QT prolongation associated with ventricular arrhythmias including torsades de pointes, requiring electrical cardioversion after amiodarone administration, after she had been drinking large quantities of among others grapefruit juice.^15^ Perhaps we should have included these references in our previous article to emphasise the fact that the interaction between grapefruit juice and amiodarone is more elaborate than previously thought. We thank the author for pointing out the typing errors in our references.
